# 3,11-Dibromo-14-(4-chloro­phen­yl)-14*H*-dibenzo[*a*,*j*]xanthene dimethyl­formamide monosolvate

**DOI:** 10.1107/S1600536812016200

**Published:** 2012-04-21

**Authors:** Yong Bin Song, Bo Liu

**Affiliations:** a Key Laboratory of Green Chemical Technology of College of Heilongjiang Province, School of Chemical and Environmental Engineering, Harbin University of Science and Technology, Harbin, People’s Republic of China

## Abstract

In the title compound, C_27_H_15_Br_2_ClO·C_3_H_7_NO, the xanthene moiety has a flattened boat conformation with a folding angle between the naphthalene units of 9.46 (3)°. The mean planes of the xanthene system and its 4-chloro­phenyl substituent are nearly perpendicular [dihedral angle = 89.43 (5)°]. The dimethyl­formamide solvent mol­ecule is disordered over two sets of sites with an occupancy ratio of 0.520 (11):0.480 (11).

## Related literature

For related structures and the preparation of the title compound, see: Wu *et al.* (2009 ▶); Seethalakshmi *et al.* (2006 ▶). For the biological activity of benzoxanthene derivatives, see: Lambert *et al.* (1997 ▶); Hideo (1981 ▶); Poupelin *et al.* (1978 ▶). For related structures, see: Cai *et al.* (2009 ▶); Lu *et al.* (2008 ▶); Rahmani *et al.* (2009 ▶); Dalla Via *et al.* (2008 ▶); Gaurrand *et al.* (2006 ▶); Petit *et al.* (2007 ▶).
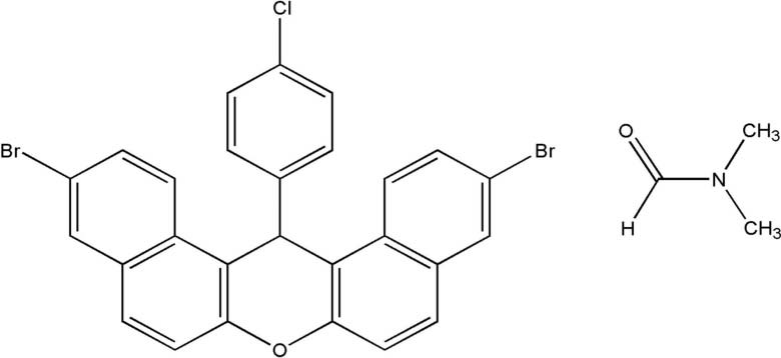


## Experimental

### 

#### Crystal data

C_27_H_15_Br_2_ClO·C_3_H_7_NO
*M* *_r_* = 623.74
Triclinic, 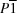

*a* = 10.8558 (12) Å
*b* = 10.9385 (12) Å
*c* = 11.8946 (13) Å
α = 74.443 (1)°
β = 80.967 (1)°
γ = 71.448 (1)°
*V* = 1286.0 (2) Å^3^
*Z* = 2
Mo *K*α radiation
μ = 3.29 mm^−1^
*T* = 293 K
0.28 × 0.26 × 0.20 mm

#### Data collection

Bruker SMART CCD area-detector diffractometer
Absorption correction: multi-scan (*SADABS*; Sheldrick, 1996 ▶) *T*_min_ = 0.415, *T*_max_ = 0.518
8696 measured reflections
4522 independent reflections
2902 reflections with *I* > 2σ(*I*)
*R*_int_ = 0.025

#### Refinement

*R*[*F* ^2^ > 2σ(*F* ^2^)] = 0.039
*wR*(*F* ^2^) = 0.091
*S* = 1.03
4522 reflections
375 parameters
55 restraints
H-atom parameters constrained
Δρ_max_ = 0.41 e Å^−3^
Δρ_min_ = −0.52 e Å^−3^

### 

Data collection: *SMART* (Bruker, 2004 ▶); cell refinement: *SAINT* (Bruker, 2004 ▶); data reduction: *SAINT*; program(s) used to solve structure: *SHELXS97* (Sheldrick, 2008 ▶); program(s) used to refine structure: *SHELXL97* (Sheldrick, 2008 ▶); molecular graphics: *SHELXTL* (Sheldrick, 2008 ▶); software used to prepare material for publication: *SHELXTL*.

## Supplementary Material

Crystal structure: contains datablock(s) I, global. DOI: 10.1107/S1600536812016200/ld2054sup1.cif

Structure factors: contains datablock(s) I. DOI: 10.1107/S1600536812016200/ld2054Isup2.hkl

Supplementary material file. DOI: 10.1107/S1600536812016200/ld2054Isup3.cml

Additional supplementary materials:  crystallographic information; 3D view; checkCIF report

## Figures and Tables

**Fig. 1.**
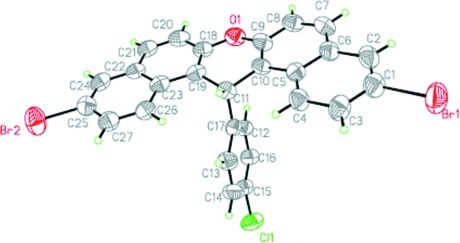
The molecular structure of the title compound, showing the atom-numbering scheme and displacement ellipsoids drawn at the 50% probability level. The solvent molecule have been omitted for clarity.

**Fig. 2.**
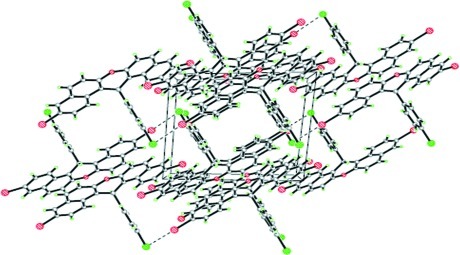
The crystal packing of the title compound viewed along the b axis. The solvent molecules have been omitted for clarity. Short Cl···Br contacts are shown by dashed lines (see Comments).

## supplementary crystallographic information

### Comment

Derivatives of benzoxanthenes have received much attention due to their wide range of biological and pharmacological activities, such as antiviral (Lambert *et al.*, 1997), antibacterial (Hideo, 1981), and anti-inflammatory (Poupelin *et al.*, 1978). In the present paper we describe the crystal structure of the title compound.

The molecular structure of the compound is shown in the Figure 1. The chlorophenyl substituent (C12–C17) at C11 forms dihedral angle of 89.43 (5)° with the mean plane of the xanthene ring system. The pyran ring (O1/C9/C10/C11/C18/C19) adopts a boat conformation with the O1 and C11 displaced by 0.112 and 0.253 (4) Å, respectively, from the mean plane of the rest of the atoms.

The packing is characterized by Cl···Br contacts and π···π stacking interactions. The distance between the Cl and Br atoms is 3.5668 (9) Å; The angles C1—Br1···Cl1 and C15—Cl1···Br1 are 161.43 (1)° and 85.97 (1)°, respectively. Some π···π stacking interactions between phenyl rings (containing Br1 and Br2, respectively) were detected with the centroid-to-centroid distance of 3.688 (2) Å. Short C-H···O contacts take place between the title molecule and the solvent.

### Experimental

A solution of the 6-bromo-2-naphthol (2.2 g, 10 mmol), and 4-chloro-benzaldehyde (0.7 g, 5 mmol), acetic acid (5 ml) was refluxed with 1 ml of hydrochloric acid for two hours (Wu *et al.*, 2009). The system was cooled to room temperature, and the formed precipitate was filtered and washed with water. The product was recrystallized from the mixed solution of ethanol and dimethylformamide (DMF), and yielded raw crystals (2.2 g, yield 81%). The colourless single crystals of the title compound were grown by recystallization from DMF solution.

### Refinement

The solvate DMF molecule is disordered over two positions. H atoms were positioned geometrically [C—H = 0.93 Å for aromatic ring, C—H = 0.98 Å for methenyl group, C—H = 0.93 Å for aldehyde group (DMF) and C—H = 0.96 Å for methyl group (DMF)] and constrained to ride on their parent atoms, with *U*_iso_(H) = *xU*_eq_(C), where *x* = 1.5 for methyl and *x* = 1.2 for all other H atoms. Positions of H atoms of Me groups were optimized rotationally.

### Crystal data

**Table:**

C_27_H_15_Br_2_ClO·C_3_H_7_NO	*Z* = 2
*M**_r_* = 623.74	*F*(000) = 624.0
Triclinic, *P*1	*D*_x_ = 1.611 Mg m^−^^3^
Hall symbol: -P 1	Mo *K*α radiation, λ = 0.71073 Å
*a* = 10.8558 (12) Å	Cell parameters from 2346 reflections
*b* = 10.9385 (12) Å	θ = 2.4–23.0°
*c* = 11.8946 (13) Å	µ = 3.29 mm^−^^1^
α = 74.443 (1)°	*T* = 293 K
β = 80.967 (1)°	Block, colourless
γ = 71.448 (1)°	0.28 × 0.26 × 0.20 mm
*V* = 1286.0 (2) Å^3^	

### Data collection

**Table:**

Bruker SMART CCD area-detector diffractometer	4522 independent reflections
Radiation source: fine-focus sealed tube	2902 reflections with *I* > 2σ(*I*)
Graphite monochromator	*R*_int_ = 0.025
phi and ω scans	θ_max_ = 25.0°, θ_min_ = 2.4°
Absorption correction: multi-scan (*SADABS*; Sheldrick, 1996)	*h* = 12→12
*T*_min_ = 0.415, *T*_max_ = 0.518	*k* = 13→13
8696 measured reflections	*l* = 14→14

### Refinement

**Table:**

Refinement on *F*^2^	Primary atom site location: structure-invariant direct methods
Least-squares matrix: full	Secondary atom site location: difference Fourier map
*R*[*F*^2^ > 2σ(*F*^2^)] = 0.039	Hydrogen site location: inferred from neighbouring sites
*wR*(*F*^2^) = 0.091	H-atom parameters constrained
*S* = 1.03	*w* = 1/[σ^2^(*F*_o_^2^) + (0.0445*P*)^2^ + 0.0361*P*] where *P* = (*F*_o_^2^ + 2*F*_c_^2^)/3
4522 reflections	(Δ/σ)_max_ = 0.001
375 parameters	Δρ_max_ = 0.41 e Å^−^^3^
55 restraints	Δρ_min_ = −0.52 e Å^−^^3^

### Special details

**Table:**

Geometry. All e.s.d.'s (except the e.s.d. in the dihedral angle between two l.s. planes) are estimated using the full covariance matrix. The cell e.s.d.'s are taken into account individually in the estimation of e.s.d.'s in distances, angles and torsion angles; correlations between e.s.d.'s in cell parameters are only used when they are defined by crystal symmetry. An approximate (isotropic) treatment of cell e.s.d.'s is used for estimating e.s.d.'s involving l.s. planes.
Refinement. Refinement of *F*^2^ against ALL reflections. The weighted *R*-factor *wR* and goodness of fit *S* are based on *F*^2^, conventional *R*-factors *R* are based on *F*, with *F* set to zero for negative *F*^2^. The threshold expression of *F*^2^ > σ(*F*^2^) is used only for calculating *R*-factors(gt) *etc*. and is not relevant to the choice of reflections for refinement. *R*-factors based on *F*^2^ are statistically about twice as large as those based on *F*, and *R*- factors based on ALL data will be even larger.

### Fractional atomic coordinates and isotropic or equivalent isotropic displacement parameters (Å^2^)

**Table:**

	*x*	*y*	*z*	*U*_iso_*/*U*_eq_	Occ. (<1)
Br1	0.52851 (4)	0.29861 (5)	0.12824 (4)	0.08221 (18)	
Br2	1.12806 (5)	−0.43299 (4)	1.11655 (4)	0.08279 (18)	
Cl1	0.34543 (9)	0.24873 (11)	0.92900 (9)	0.0742 (3)	
C1	0.6372 (3)	0.2652 (4)	0.2508 (3)	0.0561 (9)	
C2	0.7071 (4)	0.3483 (4)	0.2477 (3)	0.0568 (10)	
H2	0.7036	0.4213	0.1850	0.068*	
C3	0.6406 (3)	0.1539 (4)	0.3421 (3)	0.0565 (9)	
H3	0.5926	0.0973	0.3412	0.068*	
C4	0.7150 (3)	0.1283 (3)	0.4333 (3)	0.0508 (9)	
H4	0.7166	0.0541	0.4943	0.061*	
C5	0.7893 (3)	0.2122 (3)	0.4366 (3)	0.0440 (8)	
C6	0.7857 (3)	0.3240 (3)	0.3399 (3)	0.0508 (9)	
C7	0.8624 (4)	0.4075 (4)	0.3393 (3)	0.0587 (10)	
H7	0.8626	0.4792	0.2761	0.070*	
C8	0.9352 (4)	0.3849 (4)	0.4288 (3)	0.0571 (10)	
H8	0.9860	0.4400	0.4267	0.068*	
C9	0.9339 (3)	0.2778 (4)	0.5253 (3)	0.0507 (9)	
C10	0.8637 (3)	0.1914 (3)	0.5327 (3)	0.0443 (8)	
C11	0.8579 (3)	0.0831 (3)	0.6435 (3)	0.0438 (8)	
H11	0.8662	0.0007	0.6211	0.053*	
C12	0.7273 (3)	0.1214 (3)	0.7159 (3)	0.0394 (7)	
C13	0.6464 (3)	0.0415 (3)	0.7511 (3)	0.0517 (9)	
H13	0.6697	−0.0390	0.7295	0.062*	
C14	0.5302 (3)	0.0793 (4)	0.8187 (3)	0.0591 (10)	
H14	0.4768	0.0238	0.8432	0.071*	
C15	0.4951 (3)	0.1983 (4)	0.8487 (3)	0.0477 (8)	
C16	0.5733 (3)	0.2804 (3)	0.8162 (3)	0.0505 (9)	
H16	0.5489	0.3611	0.8375	0.061*	
C17	0.6901 (3)	0.2400 (3)	0.7506 (3)	0.0480 (8)	
H17	0.7448	0.2942	0.7294	0.058*	
C18	1.0316 (3)	0.1546 (3)	0.7020 (3)	0.0476 (8)	
C19	0.9692 (3)	0.0601 (3)	0.7161 (3)	0.0432 (8)	
C20	1.1238 (3)	0.1442 (4)	0.7777 (3)	0.0547 (9)	
H20	1.1634	0.2111	0.7652	0.066*	
C21	1.1551 (3)	0.0378 (4)	0.8683 (3)	0.0509 (9)	
H21	1.2141	0.0331	0.9193	0.061*	
C22	1.0987 (3)	−0.0661 (3)	0.8857 (3)	0.0449 (8)	
C23	1.0052 (3)	−0.0563 (3)	0.8097 (3)	0.0441 (8)	
C24	1.1331 (3)	−0.1794 (4)	0.9783 (3)	0.0517 (9)	
H24	1.1934	−0.1859	1.0288	0.062*	
C25	1.0783 (3)	−0.2792 (3)	0.9939 (3)	0.0520 (9)	
C26	0.9516 (3)	−0.1635 (3)	0.8306 (3)	0.0486 (8)	
H26	0.8905	−0.1592	0.7819	0.058*	
C27	0.9868 (3)	−0.2727 (3)	0.9200 (3)	0.0529 (9)	
H27	0.9504	−0.3419	0.9318	0.063*	
O1S	0.2639 (19)	0.1711 (15)	0.4104 (17)	0.146 (7)	0.480 (11)
N1S	0.336 (3)	0.321 (2)	0.464 (3)	0.067 (4)	0.480 (11)
C1S	0.2884 (11)	0.2997 (12)	0.3778 (11)	0.080 (3)	0.480 (11)
H1S	0.2724	0.3592	0.3057	0.096*	0.480 (11)
C2S	0.3677 (10)	0.2212 (13)	0.5620 (9)	0.091 (4)	0.480 (11)
H2SA	0.2894	0.2086	0.6071	0.136*	0.480 (11)
H2SB	0.4180	0.2444	0.6083	0.136*	0.480 (11)
H2SC	0.4179	0.1406	0.5394	0.136*	0.480 (11)
C3S	0.3956 (14)	0.4246 (12)	0.4441 (14)	0.107 (4)	0.480 (11)
H3SA	0.3941	0.4703	0.3630	0.160*	0.480 (11)
H3SB	0.4842	0.3873	0.4646	0.160*	0.480 (11)
H3SC	0.3485	0.4858	0.4915	0.160*	0.480 (11)
O1	1.0076 (2)	0.2689 (2)	0.6136 (2)	0.0576 (6)	
O1Q	0.2462 (12)	0.226 (2)	0.3776 (18)	0.174 (9)	0.520 (11)
N1Q	0.349 (3)	0.315 (3)	0.463 (3)	0.070 (4)	0.520 (11)
C1Q	0.3174 (14)	0.2003 (14)	0.4823 (11)	0.128 (5)	0.520 (11)
H1Q	0.3355	0.1260	0.5443	0.153*	0.520 (11)
C2Q	0.3047 (14)	0.4214 (14)	0.3741 (10)	0.147 (8)	0.520 (11)
H2QA	0.2946	0.3914	0.3082	0.221*	0.520 (11)
H2QB	0.3659	0.4722	0.3518	0.221*	0.520 (11)
H2QC	0.2220	0.4758	0.3994	0.221*	0.520 (11)
C3Q	0.4112 (14)	0.336 (2)	0.5499 (17)	0.196 (11)	0.520 (11)
H3QA	0.4956	0.2722	0.5586	0.294*	0.520 (11)
H3QB	0.3592	0.3261	0.6231	0.294*	0.520 (11)
H3QC	0.4208	0.4239	0.5269	0.294*	0.520 (11)

### Atomic displacement parameters (Å^2^)

**Table:**

	*U*^11^	*U*^22^	*U*^33^	*U*^12^	*U*^13^	*U*^23^
Br1	0.0733 (3)	0.1133 (4)	0.0549 (3)	−0.0121 (3)	−0.0207 (2)	−0.0202 (2)
Br2	0.1060 (4)	0.0687 (3)	0.0758 (3)	−0.0264 (3)	−0.0362 (3)	−0.0026 (2)
Cl1	0.0487 (6)	0.1130 (9)	0.0696 (7)	−0.0302 (6)	0.0088 (5)	−0.0353 (6)
C1	0.057 (2)	0.070 (3)	0.039 (2)	−0.005 (2)	−0.0072 (17)	−0.023 (2)
C2	0.063 (2)	0.060 (2)	0.038 (2)	−0.007 (2)	0.0002 (17)	−0.0135 (18)
C3	0.062 (2)	0.065 (3)	0.049 (2)	−0.0176 (19)	−0.0057 (18)	−0.024 (2)
C4	0.056 (2)	0.056 (2)	0.044 (2)	−0.0175 (18)	−0.0070 (16)	−0.0140 (17)
C5	0.0429 (19)	0.053 (2)	0.0359 (19)	−0.0108 (16)	0.0036 (14)	−0.0176 (16)
C6	0.049 (2)	0.056 (2)	0.044 (2)	−0.0094 (18)	0.0044 (16)	−0.0184 (18)
C7	0.070 (3)	0.057 (2)	0.046 (2)	−0.024 (2)	0.0081 (19)	−0.0097 (18)
C8	0.063 (2)	0.064 (2)	0.051 (2)	−0.030 (2)	0.0061 (19)	−0.017 (2)
C9	0.053 (2)	0.063 (2)	0.043 (2)	−0.0222 (19)	0.0017 (16)	−0.0205 (18)
C10	0.0443 (19)	0.050 (2)	0.041 (2)	−0.0149 (16)	0.0063 (15)	−0.0176 (16)
C11	0.0455 (19)	0.049 (2)	0.045 (2)	−0.0199 (16)	−0.0032 (15)	−0.0172 (16)
C12	0.0409 (18)	0.048 (2)	0.0365 (18)	−0.0180 (16)	−0.0092 (14)	−0.0109 (15)
C13	0.053 (2)	0.053 (2)	0.059 (2)	−0.0250 (18)	−0.0058 (17)	−0.0171 (18)
C14	0.048 (2)	0.078 (3)	0.061 (2)	−0.036 (2)	0.0011 (18)	−0.015 (2)
C15	0.0389 (19)	0.068 (2)	0.040 (2)	−0.0186 (18)	−0.0036 (14)	−0.0161 (18)
C16	0.052 (2)	0.058 (2)	0.047 (2)	−0.0193 (18)	−0.0018 (16)	−0.0182 (17)
C17	0.045 (2)	0.058 (2)	0.047 (2)	−0.0249 (17)	−0.0014 (16)	−0.0119 (17)
C18	0.044 (2)	0.056 (2)	0.051 (2)	−0.0202 (18)	0.0009 (16)	−0.0225 (18)
C19	0.0373 (18)	0.054 (2)	0.044 (2)	−0.0141 (16)	−0.0022 (14)	−0.0193 (17)
C20	0.048 (2)	0.068 (3)	0.062 (3)	−0.0268 (19)	−0.0010 (18)	−0.028 (2)
C21	0.040 (2)	0.067 (2)	0.056 (2)	−0.0183 (18)	−0.0060 (16)	−0.028 (2)
C22	0.0354 (18)	0.059 (2)	0.046 (2)	−0.0136 (17)	0.0002 (15)	−0.0223 (18)
C23	0.0359 (18)	0.054 (2)	0.048 (2)	−0.0112 (16)	0.0036 (15)	−0.0261 (17)
C24	0.041 (2)	0.068 (2)	0.049 (2)	−0.0086 (18)	−0.0058 (15)	−0.0255 (19)
C25	0.055 (2)	0.052 (2)	0.049 (2)	−0.0109 (18)	−0.0051 (17)	−0.0147 (17)
C26	0.045 (2)	0.053 (2)	0.053 (2)	−0.0129 (17)	−0.0094 (16)	−0.0198 (18)
C27	0.050 (2)	0.056 (2)	0.056 (2)	−0.0157 (18)	−0.0043 (17)	−0.0184 (19)
O1S	0.219 (18)	0.105 (8)	0.152 (11)	−0.095 (10)	0.051 (10)	−0.071 (7)
N1S	0.066 (10)	0.033 (6)	0.091 (9)	0.000 (6)	0.001 (7)	−0.017 (5)
C1S	0.066 (6)	0.090 (7)	0.071 (5)	−0.016 (5)	−0.004 (4)	−0.008 (5)
C2S	0.088 (6)	0.121 (8)	0.061 (6)	−0.047 (6)	−0.009 (5)	0.005 (5)
C3S	0.111 (8)	0.089 (7)	0.124 (9)	−0.039 (6)	0.024 (7)	−0.038 (6)
O1	0.0637 (16)	0.0636 (16)	0.0566 (16)	−0.0333 (13)	−0.0057 (12)	−0.0140 (13)
O1Q	0.058 (6)	0.28 (2)	0.247 (18)	−0.025 (9)	0.000 (8)	−0.201 (17)
N1Q	0.063 (7)	0.087 (8)	0.063 (6)	−0.032 (6)	−0.003 (5)	−0.009 (6)
C1Q	0.157 (12)	0.112 (9)	0.119 (11)	−0.055 (8)	0.039 (8)	−0.045 (8)
C2Q	0.137 (11)	0.118 (10)	0.084 (8)	0.045 (9)	0.023 (7)	0.029 (7)
C3Q	0.126 (11)	0.26 (2)	0.236 (19)	0.050 (12)	−0.093 (12)	−0.191 (18)

### Geometric parameters (Å, º)

**Table:**

Br1—C1	1.906 (4)	C19—C23	1.440 (4)
Br2—C25	1.898 (3)	C20—C21	1.350 (4)
Cl1—C15	1.756 (3)	C20—H20	0.9300
C1—C2	1.346 (5)	C21—C22	1.410 (4)
C1—C3	1.392 (5)	C21—H21	0.9300
C2—C6	1.413 (5)	C22—C24	1.411 (4)
C2—H2	0.9300	C22—C23	1.424 (4)
C3—C4	1.370 (5)	C23—C26	1.416 (4)
C3—H3	0.9300	C24—C25	1.360 (5)
C4—C5	1.413 (4)	C24—H24	0.9300
C4—H4	0.9300	C25—C27	1.400 (5)
C5—C10	1.429 (4)	C26—C27	1.363 (4)
C5—C6	1.432 (4)	C26—H26	0.9300
C6—C7	1.416 (5)	C27—H27	0.9300
C7—C8	1.347 (5)	O1S—C1S	1.453 (17)
C7—H7	0.9300	N1S—C1S	1.32 (2)
C8—C9	1.406 (5)	N1S—C2S	1.37 (3)
C8—H8	0.9300	N1S—C3S	1.42 (2)
C9—C10	1.369 (4)	C1S—H1S	0.9300
C9—O1	1.383 (4)	C2S—H2SA	0.9600
C10—C11	1.527 (4)	C2S—H2SB	0.9600
C11—C19	1.512 (4)	C2S—H2SC	0.9600
C11—C12	1.536 (4)	C3S—H3SA	0.9600
C11—H11	0.9800	C3S—H3SB	0.9600
C12—C13	1.374 (4)	C3S—H3SC	0.9600
C12—C17	1.384 (4)	O1Q—C1Q	1.485 (18)
C13—C14	1.392 (5)	N1Q—C2Q	1.36 (3)
C13—H13	0.9300	N1Q—C1Q	1.36 (2)
C14—C15	1.363 (5)	N1Q—C3Q	1.42 (2)
C14—H14	0.9300	C1Q—H1Q	0.9300
C15—C16	1.369 (4)	C2Q—H2QA	0.9600
C16—C17	1.388 (4)	C2Q—H2QB	0.9600
C16—H16	0.9300	C2Q—H2QC	0.9600
C17—H17	0.9300	C3Q—H3QA	0.9600
C18—C19	1.368 (4)	C3Q—H3QB	0.9600
C18—O1	1.381 (4)	C3Q—H3QC	0.9600
C18—C20	1.408 (5)		
			
C2—C1—C3	122.1 (3)	C19—C18—C20	122.6 (3)
C2—C1—Br1	120.1 (3)	O1—C18—C20	114.6 (3)
C3—C1—Br1	117.8 (3)	C18—C19—C23	117.5 (3)
C1—C2—C6	119.7 (3)	C18—C19—C11	121.1 (3)
C1—C2—H2	120.1	C23—C19—C11	121.2 (3)
C6—C2—H2	120.1	C21—C20—C18	120.5 (3)
C4—C3—C1	119.5 (3)	C21—C20—H20	119.8
C4—C3—H3	120.2	C18—C20—H20	119.8
C1—C3—H3	120.2	C20—C21—C22	120.2 (3)
C3—C4—C5	121.4 (3)	C20—C21—H21	119.9
C3—C4—H4	119.3	C22—C21—H21	119.9
C5—C4—H4	119.3	C21—C22—C24	120.7 (3)
C4—C5—C10	122.8 (3)	C21—C22—C23	119.7 (3)
C4—C5—C6	117.4 (3)	C24—C22—C23	119.5 (3)
C10—C5—C6	119.8 (3)	C26—C23—C22	117.6 (3)
C2—C6—C7	121.7 (3)	C26—C23—C19	123.0 (3)
C2—C6—C5	119.7 (3)	C22—C23—C19	119.4 (3)
C7—C6—C5	118.6 (3)	C25—C24—C22	120.2 (3)
C8—C7—C6	121.2 (3)	C25—C24—H24	119.9
C8—C7—H7	119.4	C22—C24—H24	119.9
C6—C7—H7	119.4	C24—C25—C27	121.5 (3)
C7—C8—C9	119.6 (4)	C24—C25—Br2	119.8 (3)
C7—C8—H8	120.2	C27—C25—Br2	118.6 (3)
C9—C8—H8	120.2	C27—C26—C23	122.1 (3)
C10—C9—O1	122.8 (3)	C27—C26—H26	119.0
C10—C9—C8	123.1 (3)	C23—C26—H26	119.0
O1—C9—C8	114.1 (3)	C26—C27—C25	119.1 (3)
C9—C10—C5	117.6 (3)	C26—C27—H27	120.4
C9—C10—C11	120.8 (3)	C25—C27—H27	120.4
C5—C10—C11	121.4 (3)	C1S—N1S—C2S	119.2 (16)
C19—C11—C10	109.9 (3)	C1S—N1S—C3S	121 (3)
C19—C11—C12	109.9 (3)	C2S—N1S—C3S	116.1 (18)
C10—C11—C12	110.6 (3)	N1S—C1S—O1S	111.3 (17)
C19—C11—H11	108.8	N1S—C1S—H1S	124.3
C10—C11—H11	108.8	O1S—C1S—H1S	124.3
C12—C11—H11	108.8	C18—O1—C9	118.2 (3)
C13—C12—C17	117.9 (3)	C2Q—N1Q—C1Q	123.1 (17)
C13—C12—C11	123.0 (3)	C2Q—N1Q—C3Q	116.9 (19)
C17—C12—C11	119.1 (3)	C1Q—N1Q—C3Q	119 (2)
C12—C13—C14	120.9 (3)	N1Q—C1Q—O1Q	100.8 (16)
C12—C13—H13	119.5	N1Q—C1Q—H1Q	129.6
C14—C13—H13	119.5	O1Q—C1Q—H1Q	129.6
C15—C14—C13	119.5 (3)	N1Q—C2Q—H2QA	109.5
C15—C14—H14	120.3	N1Q—C2Q—H2QB	109.5
C13—C14—H14	120.3	H2QA—C2Q—H2QB	109.5
C14—C15—C16	121.4 (3)	N1Q—C2Q—H2QC	109.5
C14—C15—Cl1	119.4 (3)	H2QA—C2Q—H2QC	109.5
C16—C15—Cl1	119.1 (3)	H2QB—C2Q—H2QC	109.5
C15—C16—C17	118.3 (3)	N1Q—C3Q—H3QA	109.5
C15—C16—H16	120.8	N1Q—C3Q—H3QB	109.5
C17—C16—H16	120.8	H3QA—C3Q—H3QB	109.5
C12—C17—C16	121.9 (3)	N1Q—C3Q—H3QC	109.5
C12—C17—H17	119.1	H3QA—C3Q—H3QC	109.5
C16—C17—H17	119.1	H3QB—C3Q—H3QC	109.5
C19—C18—O1	122.8 (3)		
